# Comprehensive Genomic Studies on the Cell Blocks of Pancreatic Cancer

**DOI:** 10.3390/diagnostics14090906

**Published:** 2024-04-26

**Authors:** Ricella Souza da Silva, Maria João Pina, Luís Cirnes, Luís Gouveia, André Albergaria, Fernando Schmitt

**Affiliations:** 1 IPATIMUP Diagnostics, IPATIMUP—Institute of Molecular Pathology and Immunology of Porto University, 4200-135 Porto, Portugal; ricellas@i3s.up.pt (R.S.d.S.);; 2 Faculty of Medicine, University of Porto, 4200-319 Porto, Portugal; 3 I3S—Instituto de Investigação e Inovação em Saúde, University of Porto, 4200-135 Porto, Portugal; 4 CINTESIS@RISE (Health Research Network), Faculty of Medicine, University of Porto, 4200-319 Porto, Portugal

**Keywords:** pancreatic cancer, cell blocks, cytopathology, molecular testing, large genomic panel

## Abstract

Pancreatic cancer is one of the deadliest malignancies, characterized by late-stage diagnosis and limited treatment options. Comprehensive genomic profiling plays an important role in understanding the molecular mechanisms underlying the disease and identifying potential therapeutic targets. Cell blocks (CBs), derived from EUS-FNA, have become valuable resources for diagnosis and genomic analysis. We examine the molecular profile of pancreatic ductal adenocarcinoma (PDAC) using specimens obtained from CB EUS-FNA, across a large gene panel, within the framework of next-generation sequencing (NGS). Our findings revealed that over half (55%) of PDAC CB cases provided adequate nucleic acid for next-generation sequencing, with tumor cell percentages averaging above 30%. Despite challenges such as low DNA quantification and degraded DNA, sequencing reads showed satisfactory quality control statistics, demonstrating the detection of genomic alterations. Most cases (84.6%) harbored at least one gene variant, including clinically significant gene mutation variants such as KRAS, TP53, and CDKN2A. Even at minimal concentrations, as long as the extracted DNA is of high quality, performing comprehensive molecular profiling on PDAC samples from cell blocks has remained feasible. This strategy has yielded valuable information about the diagnosis, genetic landscape, and potential therapeutic targets, aligning closely with a precision cytopathology approach.

## 1. Introduction

Pancreatic cancer is one of the deadliest malignancies, characterized by a late-stage diagnosis and limited treatment options, with a 5-year relative survival rate of <9% [1]. Among pancreatic malignancies, pancreatic ductal adenocarcinoma (PDAC) is the most common primary malignancy [2].

Surgical resection stands as the sole curative approach for PDAC, with only 20% of patients meeting the criteria for eligibility [3]. However, the diagnosis often reveals that the remaining 80% of patients present with either distant metastases or locally advanced disease, rendering them generally unresponsive to conventional therapeutic protocols [4].

For establishing pathological diagnosis in suspected pancreatic cancer, endoscopic ultrasound-guided fine-needle aspiration (EUS-FNA) is the modality of choice [5]. Pancreatobiliary cytology samples can be classified according to the Papanicolaou Society of Cytopathology (PSC) [6] and/or through the updated World Health Organization (WHO) [7] reporting system. Recent studies have suggested that the proposed WHO international system changes could more effectively stratify pancreatic neoplasms and achieve improved stratification based on the risk of malignancy (ROM) compared to the PSC system [8,9].

Despite advances in oncology over the past several decades that have improved the overall survival of patients with various cancers through the implementation of new techniques in early diagnosis, therapeutic drugs, and personalized therapy [10], this approach remains a challenge in pancreatic cancer. The advanced disease presentation impacts surgical resectability, thereby restricting the availability of surgical specimens for analysis, whether in prospective or retrospective studies [11].

In the context of PDAC, a timely identification of actionable and prognostic alterations is imperative for guiding tailored therapeutic interventions, irrespective of the tumor resectability status. Since 2019, the National Comprehensive Cancer Network (NCCN) has recommended genetic testing for patients diagnosed with pancreatic adenocarcinoma that includes universal germline testing and tumor gene profiling for metastatic, locally advanced, or recurrent disease [12].

Continued research is vital for comprehending both the clinical and molecular facets of pancreatic cancer, with the goal of advancing early detection techniques and enhancing treatment possibilities. The molecular landscape exploration and its practical application in pancreatic cancer have progressed relatively slowly compared to other cancer types, primarily due to various overlapping practical challenges related to specimen quantity and quality for analysis [11].

Cytological analysis represents the initial approach to diagnose and determine treatment strategies. The specimens collected are increasingly valuable for conducting molecular testing, including comprehensive genomic profiling. Cell blocks (CBs) obtained from fine-needle aspiration have emerged as valuable resources for these investigations. In the practical setting of pathology laboratories, CBs have been used more often than other non-formalin-fixed cytology specimens for molecular testing.

Here, we analyze the molecular profile of PDAC cases using specimens derived from CBs obtained via EUS-FNA, predominantly across a large gene panel and in the landscape of next-generation sequencing (NGS).

## 2. Materials and Methods

### 2.1. Citological Samples

CB EUS-FNA samples from 20 cases with a diagnosis of PDAC underwent targeted NGS at a reference laboratory in Portugal between January 2022 and August 2023. Clinical and cytopathological data were collected from the pathological report submitted. The NGS adequacy standards included a requirement of a tumor fraction ≥10% and a minimum of 1000 tumor cells. The overall tumor cellularity assessment of CBs relied on the evaluation of hematoxylin–eosin-stained slides, assuming that a minimum of 10 unstained slides with a thickness of 10 µm could be obtained by sectioning the CB.

### 2.2. DNA Quantification and Quality Assessment, and Mutation Testing

DNA extraction was performed using the Maxwell^®^ RSC DNA FFPE Kit (Promega, Madison, WI, USA), following the manufacturer’s instructions, which employs paramagnetic particles to purify nucleic acid. The samples were pre-processed to disrupt the cellular structure, allowing for the separation of soluble DNA from debris. The DNA then bound to the particles in a cartridge, underwent washes, and was finally eluted.

DNA quantification was performed using the Qubit^®^ 2.0 Fluorometer (Invitrogen, Waltham, MA, USA). Following the manufacturer’s instructions, we prepared Qubit™ tubes for both the standards and samples. The Qubit™ dsDNA HS Reagent was appropriately diluted in a Qubit™ dsDNA HS Buffer to create a working solution, which was then added to each tube. The standards provided by Qubit™ were introduced into the designated tubes, along with the user samples. After brief vortexing to ensure proper mixing without bubble formation, the tubes were incubated for 2 min. Subsequently, the standards and samples were read using the Qubit™ system according to standard protocols. This process ensured an accurate quantification of DNA concentrations ranging from 0.005 to 120 ng/μL, with a detection range of 0.1 to 120 ng.

A next-generation sequencing (NGS) panel was employed for the identification of somatic genomic alterations, which included SNVs, indels, CNVs, and rearrangements. This was performed with one of the following panels: Oncomine™ BRCA Research Assay, Oncomine™ Comprehensive Assay v3, Oncomine™ Focus Assay (Ion Torrent, Waltham, MA, USA), and the FoundationOne^®^ CDx (Foundation Medicine Inc., Cambridge, MA, USA) test.

The multiplex PCR-based test allows for the analysis of over 100 multi-biomarkers in solid tumors, including the most relevant driver genes for pancreatic cancer, such as *AKT1*, *ALK*, *BRAF*, *CTNNB1*, *CDKN2A*, *DDR2*, *EGFR*, *ERBB2*, *ERBB4*, *FBX7*, *FGFR3*, *FGFR1*, *FGFR2*, *KRAS*, *MAP2K1*, *MET*, *NOTCH1*, *NRAS*, *PTEN*, *PIK3CA*, *STK11*, *SMAD4*, and *TP53.*

According to the panel used, libraries were generated using 1–20 ng of DNA and/or RNA from tissue cell block sections, according to the manufacturer. The final libraries were quantified by qPCR with the Ion Library TaqMan Quantitation Kit (Ion Torrent, Waltham, MA, USA) and used for template preparation performed using the Ion Chef (Ion Torrent, Waltham, MA, USA).

### 2.3. Next-Generation Sequencing and Bioinformatic Analysis

Loaded chips were sequenced in Ion S5 or Ion S5 XL Systems (Thermo Fisher, Waltham, MA, USA). The sequencing quality was assessed through plug-in coverage analysis, and the samples were analyzed using dedicated bioinformatic workflows within the Ion Reporter v5.10 server (Ion Torrent, Waltham, MA, USA). Samples with a number of reads <100,000 and/or the average base coverage <500× were considered inadequate for analysis. The amplicons with a coverage <200× were considered non-informative. Mutations with allele frequencies of at least 5% and adequate coverage in target regions were considered to call a mutation in a patient sample. Samples with median absolute pairwise difference values less than 0.3 were considered suitable for copy number variation (CNV). Copy number gain was defined as a total copy number greater than 4.

For all the variants, the detected nomenclature was in accordance with the Human Genome Variation Society’s (HGVS) guidelines and clinical relevance accessed based on literature and/or population and disease databases. Polymorphisms, synonymous, or intronic mutations were excluded.

### 2.4. Statistical Analysis

The analysis was descriptive, with categorical data presented as absolute (n) and relative frequencies. The medians, interquartile ranges (IQR), and minimum and maximum values were calculated for continuous variables. Statistical analyses were conducted using the Statistical Package for Social Sciences (SPSS, IBM Corp, Chicago, IL, USA) software, version 25.0.

## 3. Results

The average age of the study population was 61.5 years (range 35–74 years). The CB samples were derived from 9 females (45%) and 11 (55%) males who underwent cytopathological diagnosis. Fifteen of the twenty PDAC cases (75%) had lesions located in the pancreatic head, three (15%) had them in the pancreatic neck, one (5%) in the pancreatic body, and one (5%) had them involving the pancreatic neck and body.

**Figure 1:**
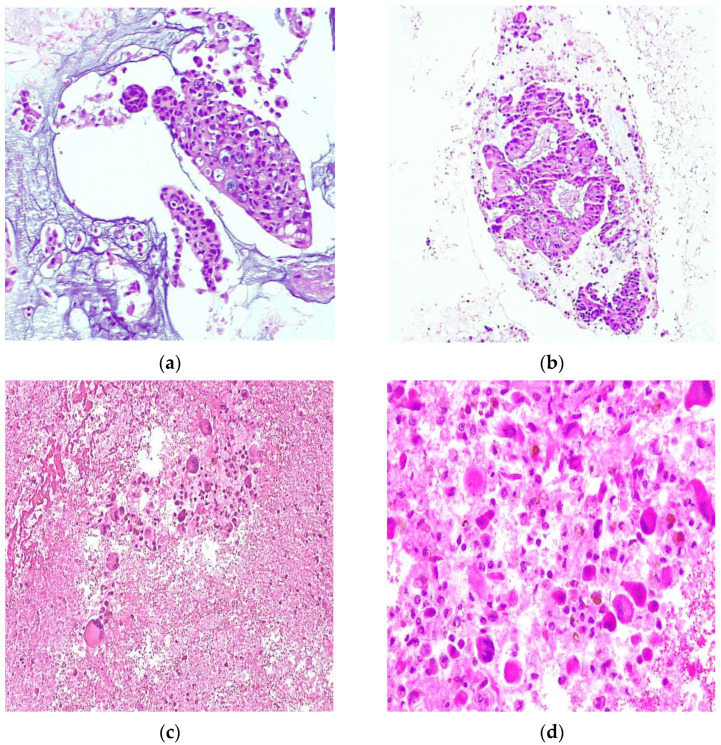
Representative PDAC cell blocks adequate for large panels by next-generation sequencing: (**a**,**b**) PDAC showing cohesive clusters of ductal cells with a moderate enlargement, nuclear hypochromasia, and a “drunken honeycomb” pattern and intercalated duct-like structure; (**c**,**d**) PDAC showing a dirty background with prominent necrotic debris, and extreme pleomorphism or anisonucleosis, with an almost complete lack of glandular differentiation were observed.

All hematoxylin–eosin-stained sections of the CBs were diagnosed as PDAC, classified as category VI, i.e., malignant according to the PSC. The cytopathological reports described cytomorphologic features consistent with conventional-type pancreatic ductal adenocarcinoma (PDAC) in 14 out of 20 cases. These features were characterized as low-grade PDAC, demonstrating cohesive clusters of ductal cells with a moderate enlargement, nuclear hypochromasia, and a “drunken honeycomb” pattern or intercalated duct-like structure. In the remaining six cases, features consistent with high-grade PDAC were identified, exhibiting poorly cohesive cells with anisonucleosis amidst a background of acute inflammatory and necrotic debris (Figure 1).

### 3.1. Evaluation of Specimen Adequacy for Molecular Testing

Thirteen of the 20 CBs (55%) were adequate for NGS. In five (71.4%) of the seven samples where the NGS test was not feasible, the percentage or absolute number of neoplastic cells was below the detection limit of the method. In the remaining two samples (28.6%), the amount of extracted DNA was insufficient, showing a high level of degradation.

**Figure 2:**
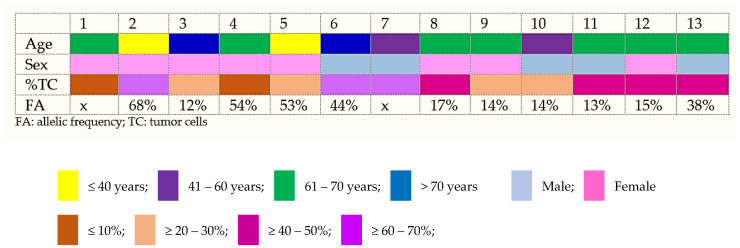
Distribution of the age, gender, percentage of tumor cells, and allelic frequency in PDAC cell block cases.

For NGS adequate samples, the median (min–max) percentage of tumor cells estimated was 36% (5–70). Of those, two samples (15.4%) presented with ≤10% of tumor cells, four samples ≥20–30% (30.8%), four (30.8%) of the samples contained ≥40–50% of tumor cells, and three (23%) of the samples contained ≥60–70% of tumor cells (Figure 2).

### 3.2. PDAC NGS Results

The 13 CBs FNA samples were sequenced using targeted specific gene mutations. According to the results obtained for DNA and RNA analyses, 11 (84.6%) of the 13 cases harbored at least one gene variant. Most samples (7/13) carried at least two clearly pathogenic mutations in different genes; there were two (15.4%) cases with five gene alterations, two (15.4%) cases with four gene alterations, one case with three gene alterations, two cases with two alterations, and two (15.4%) with no genomic alteration.

Regarding the molecular analysis of just the 22 most relevant pancreatic cancer genes, known pathogenic and/or putative driver mutations were identified in the KRAS, TP53, CDKN2A, ATM, and PIK3CA. The genes more frequently mutated were KRAS and TP53. The detection of a mutation in KRAS allowed for the molecular diagnosis of PDAC in 10 of 13 (77%) cases. As expected, most patients with a KRAS mutation (10/10) carried a missense variant at codon 12. KRAS mutations were present at an allelic frequency ranging from 14% to 44%. A TP53 mutation was detected in 4 out of 13 cases (30.8%), with variant allele frequencies greater than 10%.

Other mutations detected in this study included mutations in the genes CDKN2A (2 of 13; 15.4%), PIK3CA (1 of 13; 7.7%), ATM (1 of 13; 7.7%), RAD51C (1 of 13; 7.7%), JAK1 (1 of 13; 7.7%), NRAS (1 of 13; 7.7%), ACVR1B loss (1 of 13; 7.7%), and MTAP loss (1 of 13; 7.7%). For two cases (Foundation Medicine^®^ tests), comprehensive genomic profiling (CGP) was performed to determine the tumor mutation burden (TMB) and microsatellite instability (MS). However, the results showed no clinical significance: MS-Stable (2/2); TMB = 2 Mut/Mb (1/2); TMB = 0 Mut/Mb (1/2) (1/2). The isolated mutation assay for homologous recombination-related genes, including BRCA1/2, was negative for both cases.

An ultra-deep sequencing using NGS CGP was obtained for 11 of the 13 cases, with averages of 0.336 ng/μL DNA yields (0.142–0.596 ng/μL) and 4 ng input DNA (1.62–8.94 ng). Five (55.5%) samples exhibited “TOO LOW” DNA quantification, however with gene alterations detected for all these samples. Of these 11 cases providing good-quality DNA, an average of 2.2 million mapped reads were obtained per sample, of which 95.3% aligned with the target. The average of the sequenced read depths was approximately 2587.14 ± 1613.31, and the median was 773.9 (with a range of values from 2.613 to 5203). Overall, the target regions had a mean coverage of 1550, allowing for the robust detection of mutant alleles (see detailed description in Table 1).

## 4. Discussion

Sampling and diagnosing PDAC often relies on EUS-FNA as the primary technique [5]. While the diagnostic accuracy of cytological samples for pancreatic lesions is well established (ranging from 78% to 95%), achieving a sensitivity of 85% and a specificity of 96% [7], the incorporation of targeted NGS into routine EUS-FNA specimens from PDAC patients still holds potential in the precision medicine era.

The present study observed the feasibility of characterizing the tumor molecular profile in cytologically diagnosed PDAC patients. This was achieved by utilizing an NGS approach on CB specimens obtained via EUS-FNA and processed at different tertiary laboratories. The success of comprehensive molecular profiling varies, and uncertainties persist regarding the adequacy of the material obtained, particularly the viability of DNA extracted and the challenges of RNA extraction from pancreatic tissue due to enzymatic degradation [13].

In comparison to fine-needle biopsy (FNB) samples, studies have demonstrated similar success rates for comprehensive molecular analysis using FNA and FNB specimens of pancreatic adenocarcinoma, with complete concordance between the histologic sample and the corresponding cytologic material in almost 90% of cases [11,14]. In a study by Gan et al. (2022) [15], CBs were presumed to represent samples obtained from EUS-FNB and yield optimal material for targeted NGS in PDACs, similar to cytological smears.

However, nucleic acids extracted from CB material exhibit a lower quality compared to non-formalin-fixed cytology specimens. Despite this, CB preparations have become integral to routine surgical pathology laboratories and are frequently employed for molecular testing. This trend can be attributed to the ability of cell blocks to produce multiple sections, enabling the retention of diagnostic slides while providing material for molecular analysis [16].

Moreover, in the realm of precision medicine, molecular assays previously validated for use with formalin-fixed paraffin-embedded (FFPE) samples were also validated for application with cell blocks. If the tumor cellularity meets the minimum detection threshold of the assay and the amount of nucleic acid meets the test specifications, paraffin scrolls can be directly cut from the block and placed into a microcentrifuge tube for nucleic acid extraction. Alternatively, if these criteria are not met, unstained sections can be cut, and nucleic acids can be extracted by cell lifting or scraping from the unstained slides [17].

Our investigation revealed that over half (55%) of PDAC cell block samples provided sufficient nucleic acids for sequencing, with tumor cell percentages averaging above 30%, characterizing samples of reasonable tumor content [18]. A tumor fraction exceeding 10%–20% is the minimum acceptable threshold for molecular techniques, and to obtain a sufficient quantity of DNA for NGS, at least 1–10 ng of DNA. In cytology material samples, cell counts ranging from 100 to 2000 cells are classified as low levels, while counts between 2000 and 5000 are intermediary levels. Samples with cell counts exceeding 5000 cells are suitable for any NGS applications, including those with large panels [19]. Maintaining a minimum of 20% tumor cells is crucial to avoid false-negative molecular tests [20].

In instances of inadequate samples, the limitations observed resembled those commonly encountered with biopsy material, including low cellularity and DNA degradation. Hypocellularity and low tumor fractions, in addition to inherent preparation-related limitations, are also associated with the nature of the disease, as up to 90% of PDAC cases consist of abundant desmoplastic stroma. However, in contrast to core-needle biopsy specimens, fine-needle aspiration samples are recognized for their improved cellularity and superior NGS metrics, attributed to their naturally higher tumor fractions and absence of stromal matrix exclusion [21].

The sequencing analysis met the quality control standards, with variant allele frequencies correlating positively with tumor cellularity. These findings compared favorably with the success rates reported in other NGS studies, typically ranging from 50% to 90%, utilizing thoracic core-needle biopsy and needle aspirate specimens [22]. Even samples with a tumor content below 10%, low DNA quantification, or low allele frequency revealed gene alterations. Limiting dilution studies demonstrated detectability of the mutations present at allele frequencies as low as 0.12% [23].

We showed the successful use of a comprehensive panel covering over 100 clinically relevant genes in CB samples, supporting the diagnostic role of NGS-based profiling in clinical settings. Clinically actionable genomic lesions are found in almost 30% of pancreatic cancers [13]. In our series, we identified genomic alteration in 10 genes through NGS testing.

Multiple combinations of genetic mutations are commonly observed in PCs and can be classified as mutational activation of oncogenes, predominantly KRAS, found in >90% of pancreatic cancers; inactivation of tumor suppressor genes such as TP53, p16/CDKN2A and SMAD4; inactivation of genome maintenance genes, such as hMLH1 and MSH2 (most of these mutations are somatic aberrations), which control DNA damage repair; and alterations in genes specifically involved in the homologous recombination repair pathway, such as BRCA1 and BRCA2 (most of these mutations are germline) [24].

KRAS, TP53, and CDKN2A mutations emerged as the most prevalent and well-established genetic alterations associated with the initiation and progression of PDAC [25,26]. Research findings indicate that the presence of three or more alterations among the four principal driver mutations (KRAS, CDKN2A, SMAD4, and TP53) is correlated with a deteriorated disease-free survival (DFS) [27].

The mutation frequencies for KRAS and TP53 observed in our study aligned with the reported ranges (e.g., 70–95% for KRAS and 20–76% for TP53), and were accordance with previous investigations analyzing EUS-FNA samples for KRAS mutation analysis, ranging from 72 to 99% [28,29,30,31]. The correlation between the mutational status of KRAS and postoperative clinical outcomes remains ambiguous [32]. Quin et al. [27] reported that tumors harboring KRAS G12D mutation exhibited a poorer DFS after surgery when compared to those characterized by wild-type KRAS.

Recent research efforts have been directed toward targeting KRAS, which is the predominant somatic mutation and a key oncogenic driver in pancreatic cancer. Preclinical and clinical evidence suggests that pancreatic cancer KRAS G12D mutations may confer sensitivity to KRAS G12D-targeted, T-cell-receptor-based adoptive cell therapy, KRAS G12D small-molecule inhibitors, or SOS1 inhibitors [33]. The consistency of NGS results between CB EUS-FNA and resected tumor tissues supports the specificity and reliability of NGS in detecting PDAC [28,34].

Apart from the predominant subgroup of individuals with KRAS-mutated pancreatic cancer, focus has shifted to the KRAS wild-type population, particularly concerning molecularly guided treatments. KRAS wild-type pancreatic ductal adenocarcinoma accounts for approximately 10.7% to 12% of all PDAC cases, as reported by Philip et al. (2022) and Dorman et al. (2023) [35,36]. The findings have indicated that this subgroup exhibits an abundance of targetable alterations and includes a greater proportion of MSI-high and tumor mutational burden-high patients [35].

KRAS wild-type metastatic PDAC has been established as a unique molecular entity, for which therapeutic opportunities exist that extend beyond gene fusion events [24]. This underscores the significance of determining the KRAS mutation status of pancreatic cancer patients to identify those who are more likely to benefit from comprehensive genomic profiling.

No clinically relevant changes were observed in any of the cases tested for MSI and TMB. A systematic review encompassing 34 studies involving 8323 patients diagnosed with pancreatic ductal adenocarcinoma (PDAC), conducted by Luchini et al [37], showed that a high MSI in PDAC is rare but exists in 1–2% of cases. Compared with conventional PDAC, MSI/dMMR PDAC is strongly associated with medullary and mucinous/colloid features and a KRAS/TP53 wild-type molecular background, with more common JAK gene mutations.

Despite being investigated in a limited number of cases, our study indicates that the MSI status assessment is feasible within the comprehensive assay context and can be conducted using initial CB material. This necessity persists despite its low frequency, especially regarding the evaluation of all conceivable treatments, including anti-PD1 immunotherapies [32].

Somatic mutations occur in a higher proportion of PDACs compared to germline mutations, with over 80% of cases arising sporadically [38]. Familial pancreatic cancer comprises only 4–10% of all cases. Most familial pancreatic cancer is attributable to hereditary breast and ovarian cancer syndrome that results from germline mutations in BRCA1/2 genes and other genes such as ATM, BRIP1, CHEK2, RAD50, and RAD51C [39]. The most prevalent pancreatic cancer germline abnormalities observed involve variants in BRCA2 [36,40]. In our series, BRCA somatic and/or germline mutations were not found, while ATM and RAD51C mutations were each observed in one case.

Lincoln et al. reported that 8.1% germline variants identified at a single laboratory were missed by tumor genomic sequencing [41]. Consequently, the integration of germline testing into clinical protocols not only supports genetic counseling for family members regarding predisposition, but also informs decisions about management and tumor gene profiling strategies.

Currently, BRCA1/2, PALB2, or other homologous recombination (HR) DNA repair pathways have seen increased interest because of the possibility to treat HR-deficient tumors with DNA-damaging or HR-targeted agents, with the approved poly ADP ribose polymerase inhibitor olaparib in patients with germline BRCA-mutated metastatic PDAC [5,24]. Thus, expanding the assessment of HR-related genes to the somatic level may identify more patients eligible for targeted therapies.

Our research demonstrates the viability of conducting comprehensive molecular profiling from cell block EUS-FNA samples to identify tumor-specific gene mutations in PDAC. This approach could be used as a complementary diagnostic tool to supplement traditional cytological evaluation, as long as proper adequacy sample criteria are followed. Furthermore, multigene sequencing is a useful tool to screen for rare, potentially actionable findings.

As of the present, systematic mutational profiling for all PDAC patients in clinical practice remains limited, potentially overlooking biomarkers crucial for personalized therapeutic approaches. The current targeted therapies for pancreatic cancer include PARP inhibitors in patients with metastatic pancreatic cancer, germline BRCA mutations, and whose disease is stable or responsive to platinum-based chemotherapy; NTRK inhibitors; and in patients with MSI-H/dMMR pancreatic tumors, pembrolizumab can be proposed as second- or later-line treatment [5,24]. However, these therapies are applicable to only a small subset of patients with pancreatic cancer, highlighting the need for further advancements to expand the target population and address the challenges faced by physicians and patients in managing this disease. In their study, Taghizadeh et al. [42] found that 28% of pancreatic adenocarcinoma patients were able to receive targeted therapy through a clinical trial.

Strategies to expand the availability of targeted therapies for pancreatic cancer patients in the future involve initiating CGP at the earliest opportunity, and identifying eligible patients who are motivated and capable of adhering to targeted treatment regimens. Moreover, promoting close collaboration between multidisciplinary tumor boards and early-phase clinical trial units [36], as well more ambitious approaches, includes establishing a dedicated pancreatic cancer center and implementing a precision oncology program [43].

Furthermore, considering that only a minority of pancreatic cancer patients (20%) are eligible for surgery, performing NGS on cytological material obtained via EUS-FNA presents a cost-effective alternative to repeating the procedure [22]. This method allows for the acquisition of diagnostic and molecular information in conclusive cases and particularly in instances where initial samples are inconclusive. The introduction of this workflow clearly has the potential to shorten response times. In our series, analyzing the comprehensive genomic profiling (CMP) assay from CB samples has demonstrated to be a suitable routine clinical practice in a molecular diagnostic laboratory.

We acknowledge certain limitations in this study, including the small sample size. Additionally, the number of samples that provided sufficient nucleic acids for sequencing may have been affected by the lack of control and standardization in the preparation of the cell block specimens, as samples were received from different tertiary laboratories. Despite this characteristic, which reflects the real-world scenario in many countries, the sequencing analysis met the quality control standards.

In instances where high-quality DNA was obtained, even at extremely low concentrations, a CMP study on CB PDAC samples was feasible, leading to a substantial detection of genomic alterations across cytological specimens. Notably, all patients who underwent tumor gene profiling had an adequate sample to produce a result.

Additionally, therapeutic approaches are continually evolving, with a significant rise in the number of approved targeted therapies observed in recent years. Presently, many patients require CGP for potential therapeutic benefits, albeit only a small fraction may benefit. Nevertheless, the impact for these individuals could be substantial in the future. This is particularly critical in malignancies with a significant unmet need for novel therapies, such as pancreatic cancer, where understanding the genetic landscape and clinical trials exploring innovative treatment modalities is imperative.

Cell blocks are frequently employed for specimen processing and our results confirm the viability of conducting CMP from these preparations in PDAC patients. This approach enables diagnosis and access to insightful management information, and is possible with the advancement of precision cytopathology.

## Figures and Tables

**Table 1:** Ultra-deep sequencing analysis with NGS across PDAC cases.

Cases	%TC	DNA Yield, ng/μL	Input DNA, ng	NGS Panel	Genes Alteration	Variant Name	Chromos. no.	Position (hg 19)	Type	Variant Annotation/Variant Effect	Variant Allele Fraction	Amino Acid Change	Coverage	Exon	DNA				RNA	
															Mapped Reads	On Target	Mean Depth	Uniformity	Mapped Reads	On Target
1	5%	0.142	2.24	Oncomine™ BRCA RA	X	X	X	X	X	X	X	X	X	X	603,223	99.26%	2.63	99.58%	NA	NA
2	60%	TOO LOW *		Oncomine™ CA v3	KRAS	c.35G>T p.(Gly12Val)	12	chr12:25398284	SNV	missense	39.36	p.Gly12Val	1992	2	3,283,028	97.02%	892.7	76.90%	NA	NA
					TP53	c.388C>T p.(Leu130Phe)	17	chr17:7578542	SNV	missense	99.56	p.Leu130Phe	229	5						
					RAD51C	c.709C>T p.(Arg237Ter)	17	chr17:56787223	SNV	nonsense	68.92	p.Arg237Ter	637	5						
3	20%	TOO LOW *		Oncomine™ CA v3	KRAS	c.34G>C p.(Gly12Arg)	12	chr12:25398284	SNV	missense	12.57	p.Gly12Arg	1520	2	3,503,790	89.13%	843.9	86.25%	NA	NA
4	10%	0.216	1.62	Oncomine™ CA v3	KRAS	c.35G>T p.(Gly12Val)	12	chr12:25398284	SNV	missense	33.05	p.Gly12Val	1652	2	2,354,334	95.62%	655.3	92.11%	NA	NA
					FANCA	c.923G>A p.(Gly308Asp)	16	chr16:89862397	SNV	missense	54.17	p.Gly308Asp	216	11						
					TP53	c.234dup p.(Ala79Serfs * 70)	17	chr17:7579452	INDEL	Frameshift Insertion	33.56	p.Ala79fs	298	4						
					ATM	c.2804C>T p.(Thr935Met)	11	chr11:108139302	SNV	missense	49.28	p.Thr935Met	828	18						
5	20%	0.346	2.60	Oncomine™ CA v3	KRAS	c.35G>T p.(Gly12Val)	12	chr12:25398284	SNV	missense	21.64	p.Gly12Val	1996	2	9,660,836	95.25%	2.613	87.27%	NA	NA
					FANCA	c.1844C>G p.(Pro615Arg)	16	chr16:89842206	SNV	missense	54.63	p.Pro615Arg	1966	21						
					ATR	c.2704T>C p.(Ser902Pro)	3	chr3:142272170	SNV	missense	53.1	p.Ser902Pro	2000	13						
					TP53	c.574C>T p.(Gln192Ter)	17	chr17:7578275	SNV	nonsense	19.65	p.Gln192Ter	682	6						
6	70%	0.448	5.38	Oncomine™ FA	KRAS	c.35G>T p.(Gly12Val)	12	chr12:25398284	SNV	missense	44	p.Gly12Val	1988	2	890,131	88.42%	2756	86.74%	73,047	95.61%
					PIK3CA	c.2078G>A p.(Arg693His)	3	chr3:178938836	SNV	missense	6	p.Arg693His	2000	14						
7	70%	0.596	8.94	Oncomine™ BRCA RA	X	X	X		X	X	X	X	X	X	1,155,958	99.51%	5203	98.26%	NA	NA
8	40%	TOO LOW *		Oncomine™ FA	KRAS	c.35G>T p.(Gly12Val)	12	chr12:25398284	SNV	missense	17	p.Gly12Val	1988	2	1,361,477	96.50%	4.652	92.24%	292,911	84.88%
9	20%	TOO LOW *		Oncomine™ FA	KRAS	c.35G>T p.(Gly12Val)	12	chr12:25398284	SNV	missense	14	p.Gly12Val	1999	2	1,332,844	98.03%	4695	94.05%	213,722	87.67%
10	30%	0.268	3.22	Oncomine™ FA	KRAS	c.35G>T p.(Gly12Val)	12	chr12:25398284	SNV	missense	14	p.Gly12Val	1999	2	199,900	97.57%	703.9	94.96%	30,188	85.86%
11	40%	TOO LOW *	4.00	Oncomine™ FA	NRAS	c.182A>G p.(Gln61Arg)	1	chr1:115256529	SNV	missense	13	p.Gln61Arg	1998	3	353,198	92.28%	1125	93.02%	34,968	67.76%
					JAK1	c.1978G>A p.(Asp660Asn)	1	chr1:65312339	SNV	missense	5	p.Asp660Asn	734	14						

CA: comprehensive assay; focus assay; NGG: next-generation sequencing; RA: research assay; TC: tumor cells. * The assay is highly selective for double-stranded DNA (dsDNA) over RNA, and is accurate for initial sample concentrations from 10 pg/µL to 100 ng/µL.

## Data Availability

The data presented in this study are available on request from the corresponding author.
